# Supplement to Clinical Report on Dysentery

**Published:** 1853-10

**Authors:** Austin Flint


### BUFFALO MEDICAL JOURNAL

AND

### MONTHLY REVIEW.

VOL. 9. OCTOBER, 1853. NO. 5.

### ORIGINAL COMMUNICATIONS.

### ART. I.—*Supplement to Clinical Report on Dysentery.*

By Austin Flint, M. D.

*Morbid Anatomy of Dysentery.* Prior to offering some remarks on the pathology, causation and management of acute dysentery, which I propose shall form a supplement to the clinical report published in several successive numbers of the Buffalo Medical Journal, the morbid anatomy of the disease claims consideration. The important bearing of the abnormal appearances disclosed by dissection on the study of most diseases, in the several aspects just named, needs no comment, and there are some special reasons why it is desirable, before entering on a discussion of the topics which will subsequently come up, to consider the post-mortem changes pertaining to dysentery. Knowledge of these changes will enable us to connect with them, and thereby better understand some of the more important of the facts developed by means of clinical observations; and it is absolutely essential as a basis of correct notions respecting the pathology, causation and management of the disease.

The opportunities afforded by the cases which I have analyzed, and others that have come under my own observation, for the acquisition of facts pertaining to the morbid appearances in dysentery, were quite limited. The whole number of cases proving fatal is not large, and an examination, post-mortem, was practicable in only a portion of this number. For contributions to our knowledge of the morbid anatomy of this, as of other diseases, we must look mainly to the labors of persons connected with hospitals where the number of cases received is sufficiently large to supply ample material, and, at the same time, every facility furnished for the study of the phenomena of the dead-house. Pre-eminently such an institution is the Imperial Royal General Hospital of the Austrian capital, (Vienna,) containing 104 wards, receiving, in a single year, 20,545 patients, of whom 2678 died. The professorship of Pathological Anatomy, in connection with this hospital, is held by Dr. Carl Rokitansky, who may, perhaps without invidiousness, be said to be the most distinguished of morbid anatomists now living. As the highest authority on any subject within the range of this department of medical science, and from the very great advantages for observation which he enjoys,* his account of the morbid appearances in dysentery commands entire confidence in its completeness and accuracy. I shall accordingly copy the section of his treatise on pathological anatomy devoted to a description of these appearances. I am led to do so the more because a translation of this treatise not having as yet been published in this country, it is accessible to comparatively but few members of the profession. A portion only of the entire work has, as yet, been prepared by the author. This portion, consisting of three volumes, has been translated, and issued among the volumes published annually by the London Sydenham Society. Its circulation is therefore limited to the subscribers to the publications of that society. The following extract from the second volume† of the work, contains Rokitansky's description of what he terms the *dysenteric process,* without curtailment or condensation:

"*The dysenteric process.* We are acquainted with the dysenteric process as a substantive‡ disease of the mucous membrane of the colon, and

---

*"Rokitansky differs from all other pathologists, in not engaging in the study or treatment of disease during life; he is not a practical physician, and seldom sees one of the many hundreds of cases whose bodies he dissects."—*Preface to Manual of Pathological Anatomy by English translator.*

† Published in 1849.

‡ The term *substantive,* as thus used by Rokitansky, and some other writers, of late, is presumed to signify an affection which, so far as our actual knowledge goes, is the chief fundamental local manifestation of the disease, not an affection secondary and symptomatic of some other one.

inasmuch as it is here presented in its most exquisite form, its habitat has been correctly fixed ever since the days of Hippocrates.

"The dysenteric process is divisible into four natural degrees or forms.

"In the lowest degree, the mucous membrane commonly presents a layer of a thin secretion, of a dirty gray and reddish color, underneath which, certain parts, commonly the projecting folds of the mucous membrane, are reddened and swollen. In this manner striæ are produced, which more or less encircle the intestine. The epithelium is either raised in the shape of small vesicles which contain clear serum, or it forms a grayish white layer, resembling the mealy scurf of the epidermis, an appearance which probably induced Linnæus to term dysentery *scabies intestinorum interna.* The subjacent mucous membrane seems excoriated, slight pressure induces hemorrhage, and it may be easily detached in the shape of a light red sanguineous pulp; its submucous cellular tissue appears infiltrated.

"The anatomical characters may be summed up as—swelling, injection and reddening, softening, (red and bleeding,) serous exudation in the shape of a delicate vesicular eruption, and consequent branny desquamation of the epidermis, (epithelium?)

"In the second degree, the textural alterations are not limited in the manner described, but extend over a larger surface, still, however, presenting a greater development at one part than at another. The mucous membrane is invested to the same extent, by a dirty gray layer, consisting of desquamated epithelium, and a thick glutinous exudation; or this may have been already removed, and the subjacent mucous membrane, in either case, appears converted into a soft, sanguineous, pale red and yellowish gelatinous substance, which may be easily detached. The internal surface of the intestine commonly presents more or less numerous protuberances, which closer examination proves to consist of a very copious infiltration of the submucous cellular (areolar) tissue; these projections or tumors were first observed by Hewson and Pringle; other authors speak of them as warty tubercular swellings, or fungoid excrescences, and M. Gély has lately termed them *hypertrophie mamelonnée du tissue sousmuqueux.* They correspond to those points of the mucous membrane at which the morbid affection is most developed; with the exception of slight redness and intumescence, especially in the circumference of the follicles, an increase in the mucous secretion, and a slight desquamation of epithelium, the intervening parts of the mucous membrane do not generally offer any marked textural changes. The entire portion of intestine is generally in a state of passive dilatation; it is distended with gas and with a dirty brown fluid, which consists of the most different materials, such as intestinal secretions, epithelium, lymph, blood and fæces; its coats are thickened, and the submucous tissue particularly is in a state of tumefaction. At this stage we meet with the laminated and tubular coagula in the evacuations, described by ancient and modern authors, especially if the exudation be of a more plastic character.

"Occasionally the affection of the follicles predominates and is accompanied by irritation, exhausting secretions, and softening: these probably constitute the characteristic signs of the so-called catarrhal or white dysentery, but which, in an anatomical point of view, is the same follicular affection of the colon as that which we have already described as accompanying chronic diarrhœa.

"In the third stage, we find the protuberances more closely set, so as to produce an uneven, lobulated appearance. The mucous membrane that invests these protuberances partly retains the above described conformation; in part it is converted into a slough, which is here and there blended with the desquamated epithelium and the exudation, and is firmly attached to them; it is of a dark-red or blackish-brown, suggilated, or grayish-green color; or the mucous membrane may have disappeared, so as to expose the infiltrated submucous cellular tissue to which the remnants of the mucous membrane remain attached in the shape of solitary, dark-red, flaccid, and bleeding, vascular tufts, or as dilated follicles, which are easily removed. The interstices of the mucous membrane are the seat of the affection in a lower degree. The protuberances occasionally are found to have coalesced, and the intestine then presents an uneven plicated surface, accompanied by an equable degree of infiltration and thickening of its parietes; the mucous membrane is uniformly affected over a large extent, and there are no free interstices.

"The contents of the intestine are of a dirty-brown or reddish, ichorous, fetid, flocculent and grumous character.

"In the fourth and highest degree, the mucous membrane degenerates into a black, friable, carbonized mass, which may often be subsequently voided in the shape of tubular laminæ, (so-called mortification of the mucous membrane.) The submucous cellular tissue appears to be previously infiltrated with carbonified blood, or a sero-sanguinolent fluid; or it is pallid, and the blood contained in its vessels is converted into a black solid or pulverulent mass; subsequently it shows a purulent infiltration, in consequence of the reactive inflammation which is induced in the lower healthy strata, for the purpose of eliminating the gangrenous portions.

"The affected portion of intestine, which contains a putrid, brownish-black fluid, resembling coffee grounds, may appear in a state of passive dilatation, as above described, but it is much more frequently collapsed; and if the two highest degrees continue for any length of time, the muscular coat will be reduced. The tissue of the latter is condensed, pale, ashy, peculiarly elastic and friable, and analogous to the yellow fibrous tissue.

"The peritoneal coat presents, in the higher, and particularly in the highest degree of the affection, a dirty-gray discoloration, and a total absence of lustre; at intervals it presents a dilatation and injection of its capillary vessels, and is invested with a brownish, ichorous exudation; occasionally the mesocolon, and even the mesenteric laminæ that have been in contact with them, participate in the affection. This affords a means of distinguishing dysenteric disease of the intestine on its outer surface.

"The glands of the mesocolon present a corresponding tumefaction, but they are of a dark-blue color, congested, and tumefied; but we have not succeeded in detecting in them a peculiar (specific) solid morbid product, as we have in typhus.

"The mucous membrane of the colon is, as we have already observed, the seat of the dysenteric process; and we may state it as a rule that its intensity increases from the cœcal valve downward, and consequently is met with, in the most fully developed state, in the sigmoid flexure and in the rectum. It not unfrequently passes beyond the cœcal valve, toward the ileum, but is here only seen in its mildest form.

"It commonly runs an acute course, though it is frequently chronic in the milder degrees; this, however, does not materially alter its character.

"The manner in which it terminates varies:

"1. The disease is fatal, in consequence of the more or less rapid, or more or less penetrating destruction of tissue, and the coincident exhaustion.

"2. The disease may terminate in cure, if the mucous membrane has not become disorganized in the manner above described, the normal cohesion softening, and a new layer being generated under the desquamated epithelium.

"3. In the higher degrees of the disease, when disorganization has occurred in one of the above described processes, and the mucous membrane has suffered more or less extensive destruction, one of two results ensues:

"*a.* A real cure of the loss of substance, with consolidation of the abraded portions of the intestine follows; or

"*b.* The entire process assumes a low chronic form, the specific nature of the disease is lost, and we have atomic inflammation and suppuration of the intestinal coats.

"If a cure ensues, the portions of mucous membrane which were affected in a lower degree are first restored to their normal condition; between them are small patches, or more extensive spaces, with a sinuous circumference, at which the mucous membrane is deficient, and the submucous, pale, infiltrated cellular tissue is exposed. Not unfrequently we perceive attached remnants of mucous membrane adhering to these parts. The exposed submucous cellular tissue is gradually converted, as proved by cadaveric examinations at the most various periods after the cessation of dysentery, into serous tissue; this is further condensed into sero-fibrous tissue, and by it the sinuous portions of mucous membrane, at the edge of the impaired surface, are, like the isolated remnants of mucous membrane, compressed into warty, pediculated, (polypous,) prolongations, and thus the originally sinuous circumference obtains a fringed, dentated appearance. In cases in which the loss of substance is inconsiderable, the new tissue may contract so as to bring the edges of the mucous membrane into apposition with one another and with the polypous remnants of mucous membrane, and the cicatrix is then represented by a large number of aggregated warty excrescences of the mucous membrane, between which the sero-fibrous basis from which they proceed, may be detected.

"In cases of extensive destruction of substance, the approach of the edges is rendered impossible; the deeper layers of the tissue, which takes the place of the mucous membrane, is frequently condensed into fibrous bands, which form corded projections into the intestinal cavity, interlace with one another, and not unfrequently encroach upon the caliber of the intestine in the shape of valvular or annular folds, thus giving rise to a stricture in the colon of a very peculiar form. This mode of regeneration is the more remarkable, as it closely resembles that following the destruction of the œsophageal mucous membrane by mineral acids.

"In the second case the specific affection terminates after having previously given rise to more or less extensive disorganization, but without being followed by the healing process just described. The entire disease now assumes a chronic character, and appears on the residual portion of mucous membrane as chronic catarrhal inflammation, the follicles being more or less prominently affected, and suppuration occurring in the shape of sinuses and abscesses under the mucous membrane, and between the external coats of the intestine; at the same time the intestinal canal contracts, its coats assume a rusty, dark-blue color; there is occasional exacerbation of the peritoneal irritation, and the intestine becomes fixed in consequence of exudation and infiltration in its cellular sheath and its mesentery. In this case the mucous membrane is found of a dull, red color, tumefied, and invested by a copious secretion of a glairy or purulent character; the follicles, particularly those at the end of the colon, are dilated, distended by a glassy pituita, or in a state of suppuration; there are small abscesses of the size of a hemp seed or pea, under the mucous membrane, and in the cellular tissue lying between the muscular fibres. These abscesses open upon the mucous membrane by the suppurating follicles or by other minute orifices, forming fistulous passages in various directions, and penetrating into deeper parts, so as to reach the peritoneum, and there induce inflammation; or they give rise, in the vicinity of the rectum, to the formation of large abscesses, as described by Morgagni.

"The concurrent contraction of the intestinal tube probably causes in this case, also, a diminution of its caliber, but this form presents no peculiarity to distinguish it from the effect which may be produced in every case of catarrhal inflammation attended by repeated exacerbations.

"The dysenteric process occurs in its exquisite and primary form in the colon only, with the exception of the mucous membrane of the female sexual organs, where it affects the uterine mucuous membrane in the shape of the puerperal disease.

"The dysenteric process offers the greatest analogy to the corrosion of the mucous membrane produced by a caustic acid. The consequent destruction of the tissues, as well as the phenomena of reaction, present, throughout, a close resemblance in both cases, and the stricture produced in the œsophagus has no analogue but that resulting in the colon from the dysenteric affection.

"We have found a further analogy with the dysenteric process in the erodent effect produced upon the mucous membrane of the œsophagus by the gastric juice in schinous stenosis of the pylorus."

As already remarked, the foregoing account of the anatomical lesions of dysentery is selected because the author, at the present moment, may be regarded as the highest living authority on questions pertaining to morbid anatomy. It is proper to state that other observers have found the intestinal follicles more prominently the seat of morbid appearances than appears in the description by Rokitansky. This is the case with Dr. Finger of Prague, who studied the disease as it prevailed in that city, in an epidemic form, between the years 1846 and 1848. During that period not less than 231 dissections of subjects dead with dysentery, were made in the city hospital.*

* Vide Brit. and For. Med. Chir. Review, 1852, and Buffalo Med. Jour., 1852.

The question, how far the disease involves an affection of the follicles, has given rise to much discussion. The pathological bearing of this question is not unimportant. It must be admitted that the follicles may be more or less implicated, but it would seem to be established that this is not an uniform, and, therefore, not an essential element of the disease.

Dr. Finger, as well as other observers, describes, as prominent among the morbid appearances, the presence of more or less solidified fibrin, or lymph, adhering to the mucous membrane; and all agree as to the frequency of loss of substance, or ulcerations, more or less extensive, of this membrane.

An interesting, and also important inquiry relating to the intestinal lesions in dysentery, is, whether they have a special character, or are simply due to ordinary inflammation of greater or less intensity. The bearing of this inquiry on the pathology of the disease is obvious. This point is one of those pertaining to the morbid anatomy to which there will be occasion to refer in another section of this supplement. There has been a difference of opinion among writers respecting this point. Some think that the lesions are sufficiently explicable by attributing them to ordinary inflammation existing in varying grades of intensity; others that something more than ordinary inflammation is involved. With the facts before him the point is referred to the judgment of the reader. I will only add, that, in the mind of the writer, the lymphatic exudation, the frequent implication of the follicles, and the sloughing of portions of the membrane seem to denote a peculiar form of inflammation, somewhat characteristic of the disease.

Another inquiry connected with the morbid appearances is this: To what extent are they present in all cases of dysentery? The appearances, of course, can be studied only in cases which prove fatal; and they have been studied almost exclusively in cases of epidemic dysentery, since sporadic cases very rarely end fatally. Now, how far have we a right to infer that the changes found after death exist in those cases which recover, and more especially in mild sporadic cases? It is sufficiently clear that in cases in which the disease is promptly and completely arrested, or ceases, after a brief duration, of its own accord, the anatomical lesions must be slight. This fact is true of a large proportion of sporadic cases, and of a greater or less number of the cases occurring during the prevalence of epidemic dysentery. If lesions existed in these cases to any extent, it would be impossible for all symptoms of the disease to cease so quickly and completely as is frequently observed. It has been seen already that the symptoms show a marked difference between ordinary sporadic and epidemic dysentery. It is probable that a corresponding difference obtains in the local morbid changes. The view which appears to the writer most consistent with what facts we possess is, that in ordinary sporadic dysentery there exists simple inflammation of the mucous membrane of the large intestine, generally mild in intensity, frequently not extensive, and sometimes quite limited; while in grave cases of the epidemic form of the disease, the inflammation is not only more intense and extensive, but possesses a special virulence or malignancy. An analogous difference is observed in other local inflammations, for example, between pneumonia as it is ordinarily presented, and pneumonia typhoides. The consideration of this topic falls properly under the head of the pathology of dysentery.

Another important inquiry relates to the existence of anatomical lesions of other organs than the intestine in fatal cases of dysentery. On this point the researches of Dr. Finger may be cited. In 231 cases there were found other lesions, as follows:—tuberculosis in 48 cases; cancer in 48 cases; typhus (preceding or following the dysentery) in 11 cases; lobular pneumonia in 16 cases; croupous pneumonia in 11 cases; puerperal fever in 10 cases; secondary syphilis in 7 cases; Bright's disease in 9 cases; chronic bronchitis in 5, and recent bronchitis in several casess; heart disease in 3 cases; diabetes in 1 case. As to the liver it was generally pale and anæmiated; in one case there was inflammation of the vena portæ, and in not a single case was there abscess.*

These results show conclusively that the anatomical characters of dysentery are confined to the intestinal canal. The several lesions found in certain proportions of cases evidently denote the accidental coincidence of other affections, having no essential connection with the dysenteric disease. The facts with respect to the liver are commended to the notice of those practitioners who are accustomed to regard this organ as playing an important part in the pathology of the disease. It is true that in observations made in tropical countries, abscesses, and other lesions of the liver are found in a certain proportion of cases; but this only shows the more frequent occurrence of affections of this organ in these countries.

Finally, with reference, not only to the pathological character of dysentery, but to prognosis, and measures of therapeutics, it should be borne in mind that in severe cases of the disease, especially in its epidemic form, the intestinal lesions are probably always considerable, both in intensity and extent; and that in the highest grade of severity, disorganization of the affected membrane takes place in a degree rendering restoration difficult, and often hopeless.

---

* Brit. and For. Med. Chir. Review, 1852.

### REMARKS ON THE CAUSATION, PATHOLOGY, AND MANAGEMENT OF DYSENTERY.

### *Causation.*

The analysis upon which was based the clinical report preceding this supplement, developed nothing of consequence relating to the causation of dysentery. Assuming that, were the disease preceded by any obvious circumstances to which its production might be fairly attributable, they would not altogether have escaped attention in recording the histories, this negative result of the analysis is not without significance. It leads to the conclusion that dysentery is not uniformly, and indeed rarely is preceded by apparent causes. Is this a just conclusion? I have not propounded this inquiry before, because I did not wish to engage in discussions leading beyond the circle of facts developed by the analysis. If systematic treatises be consulted, it will be found that various causes have been supposed to be adequate to the production of the disease. The most prominent among these supposed causes are sudden atmospherical changes; indulgence in acid, unripe fruit; excesses in eating or drinking, and, on the other hand insufficiency of food; the effluvium from decomposing animal matter, and what is commonly known as marsh miasmata. The competency of each and all these causes to produce dysentery, especially in its epidemic form, may be doubted, from the difficulty of frequently, if not generally, tracing to them individual cases, and, on the other hand, the frequency with which they exist without giving rise to this disease. The occasional apparent connection between them and the disease denotes nothing more than coincidence, not the relation of cause and effect. This, of course, is not saying but that they may exert, like any other circumstances affecting unfavorably the organism, an auxiliary influence, either by increasing its susceptibility to disease, or giving greater activity to the efficient cause or causes. The question is, not what may contribute to the development of the disease and thus indirectly sustain a causative relation to it, but to what agency, or agencies, is the disease specifically due: in other words, what cause or causes are capable of producing this particular disease, with, or without the conjunction of other morbid influences? I think it must be admitted that the several causes that have been mentioned rest on insufficient evidence. Atmospherical changes, indulgence in acid unripe fruit, excesses in eating and drinking, and deprivations, are circumstances existing, to a greater or less extent, in all places, and in each successive year; but cases of dysentery occur in some places and years very infrequently, and at other places and years frequently, without any corresponding variations in these supposed causes. Moreover, in the majority of individual cases, patients attacked with the disease have not been more exposed to these supposed causes than hosts of others who escape; and often the disease attacks those who are much less exposed than many others. The evidence of its dependence on the effluvium of decomposing animal matter may be disposed of in a similar manner. The apparent connection in a few instances proves no more than either a coincidence, or an indirect influence. Its causation by marsh miasmata rests on evidence, if possible, still more slender. The unknown cause of periodical fevers, thus designated, exists to a very great extent, as shown by the prevalence of these fevers, without dysentery; and, in fact, dysentery, as a secondary affection, oftener accompanies typhus, than intermittents or remittents. The latter, as is well known, are singularly exempt from abdominal symptoms. The prevalence of dysentery in situations in which miasmatic influences exist is certainly not the rule, and, hence, if any connection exists, we have no right to presume that it is more than that which would apply to any agency exerting a deleterious effect on the organism, and therefore predisposing alike to other affections as well as dysentery.

Dysentery, like all diseases, must of course have its adequate cause, or causes, and if the circumstances to which it is generally attributed are found to be insufficient, the question arises, what morbid agency, or agencies, are competent to produce it? When our knowledge of the causation of numerous, and indeed of most diseases is considered, it is not surprising that the present state of medical science does not authorize a satisfactory answer to this question. But although we may not be able to specify the causes of disease, we may possibly arrive at some important conclusions respecting their character. That dysentery, especially in its epidemic form, requres for its production a *special* or *specific* cause seems susceptible of proof. This is an important point, not only as a scientific fact, but in its practical bearings. By a special or specific cause is meant a morbid agency so peculiar that under favorable circumstances it will invariably give rise to a particular kind of disease. Thus the poisons producing the different eruptive fevers we know must be each peculiar in its character, and all essentially different from each other. They severally give rise to different forms of disease, and each always to the same form. These diseases, in other words, respectively proceed from the operation of special or specific causes. Now, the evidence appears to be conclusive that dysentery, in like manner, stands in the relation of dependence to a pathogenetic principle peculiar to itself. What are the considerations which lead to this conclusion? In reply to this interrogatory the following considerations are submitted, their application not being confined to dysentery, but extending to various other diseases:

(*a.*) The absence of obvious causes properly leads to the presumption of an occult cause. In this consists the significance, already referred to, of the inability to trace cases of dysentery to any of those causes to which the disease is loosely attributed by systematic writers. In general, when we fail to discover the evidence of any causative relation between a disease and surrounding circumstances, we are to suspect an occult cause. A cause is not necessarily special because it eludes investigation, but it is undoubtedly true that a large proportion of occult causes are special in their character.

(*b.*) The fact that the prevalence of dysentery is confined to certain places and periods tends to show its dependence on a special cause. If ordinary causes alone sufficed to produce the disease, there might, it is true, be considerable variations in the extent of its prevalence, as well as in other respects, owing to corresponding differences pertaining to these causes, but these variations could hardly be so striking as they are, inasmuch as the differences in ordinary causes, at different times and places, are not correspondingly great. In the city of Buffalo, for the ten years prior to 1849, dysentery occurred very infrequently, never prevailing, to any extent, till the year just mentioned. Now the ordinary causes of disease, viz., changes of temperature, indulgence in fruits and crude vegetables, excesses, etc., were operative, to a greater or less extent, during these ten years; nor was there any increase in the operation of these causes in 1849. In fact, in that year some of these supposed causes were less operative than usual, owing to the greater prudence in the community in consequence of the prevalence of epidemic cholera. It is, then, clearly apparent that the prevalence of the disease in 1849 depended on some morbid agency superadded to those causes of disease which uniformly exist to a greater or less extent.

(*c.*) Taking into view the evidence that the disease takes place independently of ordinary causes existing in any and all seasons, the fact that it is restricted in its occurrence to a particular season, in a temperate climate, goes to show a special causation. The cases, the histories of which were collected during several years, (vide clinical report,) were found to have all occurred in the summer and autumnal months. As a general remark, diseases which are governed by laws restraining their prevalence thus to certain seasons, owe their origin to special causes. The prevalence of dysentery at certain times, places, and seasons, constitutes it either an epidemic or endemic disease; and it is probably correct to say that epidemic or endemic diseases always involve in their production causes which are special in their character.

(*d.*) If it be correct to regard the anatomical characters of the disease as involving not merely ordinary inflammation, but a peculiar process, it would be most consistent with such a view to suppose this process to require for its production a peculiar poison. It is true that processes which have a special character do take place within the organism apparently independently of any special external agency. Examples of this fact are tubercle, carcinoma, etc. This consideration would be without much force were it not that other considerations show pretty conclusively dysentery to be produced by a morbid cause originating exterior to the body. Its prevalence as an endemic or epidemic may be considered to establish sufficiently this conclusion.

(*e.*) The special origin of the disease would be rendered analogically probable by the fact of one attack exempting the individual from its subsequent recurrence. The striking results developed by the foregoing analysis with respect to this point, however, must not be considered to establish this fact.

(*f.*) The facts pertaining to the condition of health at the time of attack in cases of dysentery have some bearing on the question under consideration. As a general remark it is probably true that the ability to resist ordinary morbid causes is diminished in proportion to the feebleness of the powers of the constitution, in consequence of present or past disease. This rule does not appear to hold good with respect to special causes of disease. The latter in some instances at least, are found to affect in preference persons of good health and constitution. This is true, for example, of typhoid fever. Facts seem to show that it is true of dysentery, notwithstanding it is stated otherwise by some systematic writers.* The facts developed by the analysis which has preceded these remarks, (see sec. ninth of Clin. Report,) lead to this conclusion. So also do the facts pertaining to the morbid anatomy of dysentery derived from the dissections by Dr. Finger (see foregoing section of supplement.) This being the case, agreeably to the general law stated above, it is a fair inference therefrom that the production of dysentery involves a special cause.

Assuming the causation of dysentery to be special or specific in its character, how much farther than this can we go with our present knowledge? It is plain we are ignorant of the nature of the special cause; else, its existence would not be a matter for argumentation. We are equally ignorant of its source, of the circumstances necessary to its development, of the mode of its diffusion, and the avenues of its ingress into the organism. But in all

---

* *e. g.* Valleix.

these particulars we are not more in the dark here than in other instances in which it is not less certain that diseases are due to special causes. All that we know of the causation of dysentery beyond the fact of the existence of an unknown special cause is, that the efficiency of the latter requires an elevated temperature. This is proved by the disease occurring during the summer or autumn, and by its greater prevalence in tropical climates.

Finally, an important inquiry suggests itself having application, not to this disease only, but to all epidemic affections, or those which arise from special causes, viz: how is science to proceed in the endeavor to discover the nature and source of these special causes? This is an important topic, and it may not be considered irrelevant in this connection to devote to it a few words.

Of dysentery, as also of any disease involving external special causes, our knowledge only embraces more or less of the morbid conditions which are effects of these causes. Of the causes themselves, their nature, source, etc., as, we have seen, we know nothing. This portion of etiology remains a *terra incognita.* Scientific research has as yet scarcely made a commencement in its exploration. It is evident that we have no clue to lead us directly to the discovery of these special causes. To seek to discover them by direct means would be useless. Can nothing be done? Yes; much might be done. Let observations pertaining to topography, meteorology, etc., sufficiently comprehensive to cover all appreciable circumstances with which the causation of the disease may be supposed to have any connection, be made so extensively as to embrace numerous situations both where the disease does, and where it does not prevail. The next step is to institute a careful comparison with respect to all these particulars of the places where the disease has prevailed, with those where it has not prevailed. This comparison would show what circumstances were common to both places, *i. e.* to places where the disease had, and where it had not prevailed. It is plain that the circumstances common to both could have had no necessary connection with the causation of the disease. They are therefore to be eliminated. By this logical process of elimination, the investigation is narrowed to those particulars which are peculiar to the places in which the disease had prevailed. Now to some of these particulars it is presumable the disease sustains a relation of dependence. The next step is to institute, as respects these particulars, a comparison of numerous places where the disease had prevailed. In this comparison it is evident the circumstances to be eliminated are those which are not common to all these places, since the disease is independent of those which are found to be not uniformly present. All particulars, then, which were present in a portion only of the places, are eliminated as having had nothing to do with the causation of the disease. The investigation by this second process of elimination is narrowed to the circumstances common to all the places in which the disease had prevailed. To some of these the disease probably stands in the relation of an effect. The problem, then, is to determine from which of these comparatively few circumstances, singly or combined, does the disease derive its origin.

What the fruits of such an investigation might be, it is of course impossible to foresee. It would not be fruitless even were the results negative, for then it would be a legitimate inference that the disease involves agencies having no connection whatever with any of the phenomena observed, and thereafter scientific research need not be retarded by attention to these phenomena, which is thus shown to be superfluous. But it is hardly probable that such an investigation would be barren in positive results. If it did not lead to the disclosure of the true source of the special cause, it would determine what circumstances exert more or less agency in directing and modifying the operation of the cause in the production of its morbid effects.

It is obvious that this plan of investigation is applicable not alone to dysentery, but other epidemic or endemic affections, and, in a measure, to all diseases; and observations made with reference to the causation of one disease would be equally available for any other, so far as concerns the external circumstances which the observations embrace.

To carry this plan of investigation into execution on a scale sufficiently large, is a project yet to be achieved. To gather together the requisite data for comparisons and the processes of elimination, in addition to meteorological and other details belonging to physical science, accurate registrations of deaths and diseases are requisite. The magnitude of the undertaking is such that it is only practicable as a measure provided for by a liberal and enlightened legislation; and it is to be hoped that the time may come, when to enlarge our knowledge of diseases with a view to lessening the mortality they occasion, increasing the probabilities of life, and enhancing its value both to individuals and the commonwealth, will be considered to be a matter as appropriate and important as any of those that appeal to the deliberations and bounty of a state or the national government.*

---

* It is proper to state that the plan sketched above was submitted by the writer in his report on practical medicine made to the Am. Med. Association, in 1851. The language employed in the report is to some extent adopted.

### *Pathology.*

The view which has been taken of the causation of dysentery, determines, to some extent, the view to be taken of its pathology. The local manifestations of the disease are in a high degree marked, but is the pathology of the disease comprised in the intestinal affection? Certainly not, if a special cause be involved. After the introduction of the special cause within the organism, prior to the dysenteric manifestations, certain morbid processes must take place. In these processes consists the essential pathology of the disease. They constitute the disease, the phenomena pertaining to the intestinal affection being only its effects, or, in other words, its local expression. What is the nature, and where the seat of these primary processes? Do they have their origin in the fluids or solids? What period do they require to be completed? How is it that they stand in such an exclusive relation to an affection of the large intestine? These, and other kindred questions cannot, in the present state of medical science, be answered. To say that the processes are analogous to the chemical principle of *catalysis*, in other words to call the disease *zymotic*, is but to indulge a conjecture, however plausible it may be. As on the subject of causation we found ourselves unable to advance beyond the simple fact of the existence of a special cause, so we must here be content with the belief that the pathology involves certain inappreciable changes, the character of which is entirely unknown. The bare fact of the existence of these unknown changes is important, not in itself alone, but in its practical bearings; and it is to be considered that we are not more ignorant of the essential pathology of dysentery than of other diseases due to specific causes.

In the practice of medicine it is often hardly less important to appreciate the poverty of the science, than to avail ourselves of its resources. Good policy, therefore, not less than propriety requires that what is not known, as well as what is known, should be fairly estimated. Moreover, a just appreciation of the limits of scientific knowledge, secures a point of departure the most favorable for the development or reception of new truth.

To say that dysentery is more than a simple colonitis, involving as it does an antecedent morbid condition, of which the intestinal affection is the result, is not to depreciate the importance of the latter in itself, nor its reactions within the organism. The local lesions, albeit in a pathological sense they do not constitute the disease, but are secondary in the order of sequence, give rise to nearly all its appreciable phenomena, and the danger to life is proportionate to their magnitude. The degree and extent of these lesions are probably proportionate to the unknown changes which precede and determine them.

The elimination of fluids through the intestinal canal is undoubtedly a very important pathological element in certain cases of dysentery. Analytical investigation shows that cases characterized by copious sero-sanguinolent discharges are peculiarly severe and dangerous. These discharges contain more or less of the component principles of the blood. The blood is necessarily the seat of serious lesions in proportion to the loss of important ingredients which it sustains. Whether the escape of the blood principles be owing entirely to the intestinal lesions, or to changes in the blood itself, is a question which can only be answered conjecturally, with our present knowledge.

The fact that symptoms of the intestinal affection constitute almost the first appreciable evidences of disease in dysentery, the prior changes determining this affection not being revealed by any marked evidences of disorder in the economy, may seem to militate against the view which has been taken of its pathology. There are, however, other instances in which the essential changes preceding the local manifestations are equally latent, for examples, tubercle, and epidemic cholera. In such instances, either the inappreciable morbid processes very quickly result in the local manifestations proper to the disease, or they are not of a character to produce other marked evidences of disorder. Which of these explanations is applicable to dysentery it is impossible to say.

In the foregoing remarks on the pathology of dysentery, as in those on the subject of causation, reference has been had especially to the disease in its epidemic form. The question arises whether in sporadic, benign cases, the same view of the pathology be applicable. The idea has been already thrown out that an essential point of difference between sporadic and epidemic dysentery may consist in the former being purely a local inflammation, while the latter involvesas has been seen, antecedent constitutional changes. I can do no more than repeat this idea in the present connection. It is, of course, entirely speculative. The discussion of the point would be likely to lead to a wider range of inquiry than comports with the actual amount of our knowledge. The question would at once arise, how far are all local inflammations not produced by exterior causes acting directly on the parts inflamed, to be regarded as local? It is customary to call such inflammations *spontaneous*, but this term implies that effects occur without causation. Morbid phenomena are never spontaneous. They seem to be so, it is true, but this is because the causes and processes upon which they depend are unseen and unknown. The term spontaneous, applied to the origin of diseases, is only significant as expressing our ignorance of their causes and mode of development. Modern investigations appear to show that some inflammations, heretofore regarded as purely local, proceed from the presence of morbid materials in the blood. Those occurring in connection with rheumatism and Bright's disease may be cited as examples. How far continued investigations may succeed in unfolding the relations of local diseases to pre-existing morbid conditions of the fluids, cannot, of course, be anticipated; but it is clear that to a rational humoralism, under the guidance of analytical chemistry, we are to look for future developments pertaining to pathology and etiology, that shall dispel the darkness which now shrouds these departments of medical science.

### *Management.*

My remarks on the management of dysentery will be confined to a few general considerations, not embracing many details, nor discussing the merits, or mode of action of the various remedies recommended in systematic works on practical medicine, or in treatises devoted specially to this disease.

It is evident that the ratio of recoveries cannot be made a test of the efficacy of treatment in benign, sporadic cases of this disease. Such cases have little or no tendency to a fatal issue. Recovery is the rule, irrespective of therapeutical measures for that end. It is probable that few cases would terminate fatally even under injudicious management. But in this disease, as in most others, our science labors under the disadvantage of not possessing the results of the analysis of a series of cases in which there was no medicinal interference. Facts of this kind, which are as difficult to obtain as they are desirable, would constitute, if attainable, the true point of departure for determining collectively and severally, the value of different modes of treatment. It is, however, plain that in the form of dysentery just mentioned, the objects to be attained by remedies are chiefly limited to the relief of distressing symptoms, shortening the duration of the disease, and, possibly, arresting at once its progress.

On the other hand, it is evident that in some severe cases of epidemic dysentery in which the disease attains to a certain degree of intensity, recovery is not to be expected. Morbid anatomy teaches us that in these cases disorganization of the intestinal mucous membranes takes place to such an extent that restoration is almost impossible. In some instances, also, it is quite clear, although not so demonstrable, that, in consequence of the loss of a portion of its constituents, the blood is the seat of lesions not less fatal, if not even more so, than those which belong to the gravest cases of epidemic cholera. The tendency to death is as marked in these cases, as is an opposite tendency in those previously referred to. So strong is this tendency that, in a certain proportion of instances, it is vain to look for efficacy toward any plan of management.

The disease may be presented in every grade of severity between these opposite extremes.

It follows from these facts that in according success, or failure to medicinal treatment, it is necessary always to take into account the wide differences in the character and tendencies of the disease, so far as it is practicable to estimate them disconnected from therapeutical measures. This is an important consideration. It affords an explanation, on the one hand, of the fact that a practitioner may meet with few or no fatal cases of dysentery, without any right to assume, in consequence, any peculiar efficacy in behalf of his mode of practice; and, on the other hand, of the fact that another practitioner, whose practice may be far more judicious, is more unfortunate. A misfortune it truly is, because it has so happened that his cases were characterized by their gravity and fatal tendency. It explains, also, the fact that measures of treatment which at one time, or place, appear to be uniformly efficacious, prove to be quite otherwise at another time, or place.

The consideration should be borne in mind in comparing the results of one's own experience at different seasons, or in individual cases, and it enforces due qualification of the testimony of others. Mortuary statistics, too, are of little practical value, and may lead to error, if not considered in connection with the character of the cases analyzed. The apparent success of different modes of practice in the same epidemic, as a criterion for determining their relative merits, may be quite fallacious. During the progress of an epidemic a host of mild cases are likely to occur, together with a proportion, greater or less, of cases more or less severe. The ratio which cases of the character last referred to, bear to the former, will greatly affect the mortality in a given number of cases, irrespective of medical treatment.

The same caution, in short, is to be exercised in drawing deductions from facts pertaining to therapeutics in this disease as in some others in like manner distinguished by widely separated extremes of mildness and severity. In this respect it resembles, for example, scarlatina, a disease which, in its mildest form, has so slight tendency to a fatal issue that, as stated by Sydenham, it rarely ends otherwise than favorably save by the officiousness of the physician; but, when malignant, destroys life, often, in spite of the best directed efforts to avert that result.

Have any remedies been found to exert a specific control over dysentery? No one will answer this question affirmatively. It cannot be claimed that we are able to neutralize the special cause within the organism, or arrest the primary morbid changes which constitute the disease, by any known method of medication. There is but little opportunity for a specific mode of treatment to be available in dysentery, since the first evidences of the essential morbid condition are the manifestations of an intestinal affection which speedily reaches its maximum of intensity. There is insufficient time for the operation of a special remedy between the first indications of disease and the development of intestinal process; the latter, also, being not more amenable to abortive measures of treatment than other forms of local inflammation. It is true that in some mild cases the disease appears to be arrested. We have seen that in a small number of the cases which have been analyzed, this result apparently followed the employment of opium, alone, and in combination with calomel. But considering the small number of such cases, and the mildness of the attack, it is probably more philosophical to suppose that they illustrate an abortive tendency in the disease, rather than any specific influence of the treatment. This, however, is not to deny that the treatment exerted a certain amount of favorable influence. What are the objects of treatment in dysentery? The answer to this question involves another inquiry, viz., what are the elements of the disease which occasion its distressing symptoms, and in which consist its severity and danger? These elements are chiefly comprised in the morbid process going on in the intestine, probably conjoined, in some instances, with blood lesions from the loss of certain of the constituents of this fluid. It is plain that the essential pathological condition, in other words the inappreciable changes induced by the primary action of the special cause, destroy life, not in themselves, but by means of the secondary changes, or results, which generally pertain, for the most part, to the intestinal affection. The gravity of the disease, *ceteris paribus*, is always in proportion to the extent and intensity of this affection. This being so, the objects of treatment, it is sufficiently apparent, are to endeavor to prevent, or abate the severity of the local manifestations of the disease; to relieve the symptoms incident thereto; to obviate unfavorable events connected with them, and to sustain the system through the processes of restoration. In this enumeration of objects are contained the leading rational indications of management.

The adaptation of therapeutical measures to the fulfillment of these indications involves, *first*, rational principles; and, *second*, experimental results. To what extent the latter are capable of furnishing evidence for, or against different measures, is an important point, to which remarks already made have had reference. Great value is generally attached to the apparent influence of remedies in this, as in other diseases. Writers and practitioners usually rest the claims of the particular methods of treatment which they recommend, on experience. Such and such measures have been found eminently efficacious; others have proved inefficient, or hurtful. This is the language of those who believe that they speak from a practical knowledge which authorizes positiveness of opinion, and should command the confidence of others. Let us examine this point a little farther, bearing in mind that the examination is applicable, not less to the therapeutics of various diseases, than to dysentery. A practitioner employs a certain method of treatment in a case, or a series of cases. The disease passes through its career without exhibiting marked gravity of symptoms, ending in recovery. Now, how is it to be determined that the treatment has divested the disease of severity and danger? Plainly, to demonstrate this to be the result of the treatment, it must be known what would have been the course and character of the disease in that case, or series of cases, if the treatment had not been employed. To know this is, of course, impossible. It would be only an approximation to this knowledge to have the history of another case, or an analysis of another series of cases in which the disease had been permitted to run its career without any treatment; but even this information is not available. I say this would be but an approximation to the knowledge necessary to solve the above problem, for no case, or series of cases, can be assumed to represent, in all respects, another case, or another series of cases. The liability to err in according to the influence of remedies what really belongs to the disease itself, must be apparent on due reflection. How much of this kind of error pervades medical experience, different persons will estimate differently, according to the disposition of the mind to distrust, or credit what is not rigorously proved. On the other hand, a practitioner makes trial of certain remedial measures in a case, or a series of cases, in which the disease exhibits persisting severity, and ends fatally. He asks himself how much of this severity is to be accounted for by the default of a more effective method of treatment; and may not the remedies have even exerted a positively injurious effect, contributing directly to the issue? In this instance, he has no means of determining, demonstratively, the true answers to such questions; and in a spirit of candor, he may be led to err in attributing to his mode of practice what belongs intrinsically to the disease itself. It may seem unsatisfactory to some persons to subject medical experience to so critical an analysis as this, inasmuch as it may beget a degree of doubt which will prove inconvenient in practice; but it is due to truth that the difficulties in the way of obtaining exact knowledge in therapeutics should be fairly considered. To be satisfied with practical notions, the correctness of which is far from being established, may contribute to the comfort of medical practice, since the mind is thereby relieved of anxiety and hesitation, as well as the trouble of farther investigation, and here, in fact, is another difficulty in the way of the endeavor to arrive at the truth.

A just appreciation of the fallacies incident to experience should by no means lead to its rejection as a means of increasing therapeutical knowledge. It only enjoins the importance of receiving experimental results with caution, and due qualifications. To say that medical experience is in no degree trustworthy, would be as unphilosophical as to overlook, or depreciate the difficulties with which it is beset. The ratio of fatality, the average duration of the disease, etc., compared in different groups of cases treated by different methods, undoubtedly may lead to valuable therapeutical deductions; and a mode of numerical investigation which appears not to have received adequate attention, consists in noting the immediate apparent effects of remedies, in other words comparing, in series of cases, the symptoms directly before, and after the administration of remedies of sufficient potency to produce immediate effects either favorable or unfavorable. The ratio of fatality and the average duration, so far as they furnish evidence of the influence of treatment, exemplify the remote effects of remedies, and of the combined effects of the different remedies which enter into the treatment, not of individual measures. The direct influence of the latter can only be studied, experimentally, by means of observations embracing the phenomena immediately antecedent and consequent; and these observations, it is obvious, must be sufficiently multiplied to avoid the error of mistaking for the effects of remedies, occurrences due to mere coincidence, or to the progress of the disease.

In endeavoring to place a proper estimate on the fruits of experimental research, a broad distinction is always to be made between the general impressions purporting to be deductions from facts retained by the memory, and the results of the analytical investigation of numerous observations which have been carefully recorded. The latter may almost be said alone to constitute the basis of true experience. To say that the former is valueless, would be equivalent to a repudiation of not an inconsiderable share of prevailing opinions respecting the therapeutical operation of remedies in different diseases. It may, however, be asserted that it is by means of the numerical system of investigation these opinions are to be confirmed, or their incorrectness proved.

With the fallacies incident to the rules of therapeutics based on experimental results alone, and the present incompleteness of this kind of knowledge, diseases must be treated, to a greater or less extent, on *rational principles*, that is to say, principles belonging to general therapeutics, applied to the management of particular affections analogically, or *a priori*. Here, too, are difficulties, and liabilities to error, but a consideration of them would open up a subject too extended for present discussion.

Various methods of treatment, it is well known, have been, and are still employed in the management of dysentery. As stated at the outset, I do not propose to treat of these methods, and the remedies which they embrace, at length. I shall notice some of the more important of them.

*Blood-letting.* Of blood-letting, general or local, in this disease, I cannot speak from personal knowledge. In the sporadic cases that have fallen under my observation there have not been present any indications for this remedy, and as the disease has been presented in my practice in an epidemic form, I have been deterred from extracting blood by observing that the mode of dying was by asthenia, and that the symptoms denoted this tendency so soon as the disease assumed a grave or dangerous character. In a few instances I have regretted, during the progress of the disease, that I had not employed blood-letting at the commencement. In these instances the patients were robust, attacked in full health, and, shortly after the disease commenced, there was well-marked febrile movement, the skin being hot and dry, and the pulse having considerable development and force. The objects to be fulfilled by blood-letting obviously are, the prevention of that intensity to which the intestinal inflammation might otherwise be expected to attain, and the abatement of the intensity already attained, in either case diminishing the tendency to disorganization, or a degree of severity of the local affection dangerous to life. Reasoning by analogy it would seem that in certain cases this remedy would be as appropriate as in other diseases characterized by the presence of a high grade of inflammatory action. Ordinary sporadic dysentery, however, seldom demands it, and it is a remedy of too much potency to be needlessly employed. In a large proportion of cases of epidemic dysentery, it is inapplicable on rational principles. Cases characterized by a diffusion and intensity of inflammation sufficient to overwhelm the vital forces like an extensive burn situated on the surface of the body, hardly admit of a therapeutical measure compromising the resources of the system which will be required for the processes of restoration. It is evident, also, that after the local affection has attained to its maximum, be it greater or less in degree, the vital powers are to be sustained, rather than diminished as they must be by the detraction of blood. In short, whenever applicable, it is very early in the disease, and under the circumstances generally considered to denote the remedy in other inflammatory affections, viz., symptomatic febrile movement, with morbid increase of power in the forces carrying on the circulation, a robust constitution, and especially general polyæmia.

Keeping the objects of bleeding in view will be likely to protect against its employment in cases of such mildness that it is not required; and, on the other hand, in cases in which from the overwhelming extent and intensity of the local affection, or the duration of the disease it would be not less injudicious and attended with greater risk of injury. Certainly when we take into view the liability, in severe cases of dysentery, to rapid disorganization, and to sero-sanguinolent effusion, producing death by exhaustion, the necessity of discrimination in the use of the lancet is not less conspicuous than the importance of promptness in its use if, by means of it, we are able to guard against these results. In these remarks I have had reference mainly to general blood-letting. They will, of course, also apply measurably to local blood-letting by cupping or leeching. Leeches applied at the anus are recommended especially by French writers, in order that the blood may be abstracted as directly as possible from the vessels of the affected intestine. It is surprising that this practice has not found favor in this country, among those practitioners whose attention, in the treatment of dysentery, seems to be absorbed with the idea of congestion of the portal circle!

*Purgatives.* I have employed purgatives in but a small number of cases of dysentery. Prior to 1849, I had treated only mild sporadic cases, and finding that they uniformly did well without purging, I was led to think this kind of medication was generally not required. Purgatives of the saline class, have of late years been highly advocated, by different writers, in the medical periodicals of this country, but I have been deterred from making trial of them in consequence of the prevalence of epidemic cholera, more or less, in this city annually, since the year just mentioned, till the present year (1853.) Purgatives, as is well known, heretofore have been considered very important in the treatment of dysentery. Under the belief that the inflammation of the intestine was produced by the direct action of its morbid contents, and that hardened fecal matter, (scybala,) was a frequent source of irritation, it was deemed a rational indication to endeavor to bring about free evacuations, and to persist in the frequent repetition of cathartics during the course of the disease. Saline purgatives were recommended, many years since, by Zimmerman, and more recently, by Bretenneau, and several other writers among the French.* The sulphates of soda, and of magnesia; the tartrate of potassa and soda, and the bi-tartrate of potassa, have each been recommended as the most eligible saline preparation to be used in this disease. The testimony borne by the authors just referred to, and by many practitioners in our own country, in favor of the good effect of these remedies, is very strong, and certainly entitles them to a fair trial.

The pathological views most consistent with our present knowledge do not lead us to employ purgatives for precisely the same end to which they were heretofore directed. The morbid condition of the intestinal contents is an effect rather than a cause of the local affection. It cannot be expected by these remedies to strike at the root of the disease. But the questions arise: is not the inflamed mucous surface placed in a situation the more favorable for restoration the less in quantity are the contents of the intestine? May not the presence of intestinal contents increase the local affection directly; and, indirectly, by exciting frequent, painful efforts for its expulsion? Is it not preferable to effect at once, or within a short time, by the action of a purgative, a thorough evacuation, than to allow the accumulation and constant passage of fecal and other matters along the affected intestine? Rationally these questions are to be answered in the affirmative. They lead to philosophical explanations of the salutary operation of this class of remedies in dysentery. Assuming their utility, there are, of course, certain precautions, and restrictions in their use, which are sufficiently obvious, and it would lead into too much detail to consider them. It is easy to see that purgatives may be used to an excess in this disease, and to such an extent that their direct action on the local affection would be highly injurious.

I was strongly impressed with the belief that purgatives must be occasionally beneficial in dysentery, by the facts observed in two cases which came under my notice in the summer of 1852, neither of the cases being recorded. In these cases the dysenteric symptoms persisted for many days, notwithstanding the remedial measures adopted, till, at length, several very copious fecal evacuations occurred spontaneously, after which immediately the disease subsided very rapidly. The coincidence was very striking in these

* Valleix.

cases, and although there may be some room for the suspicion that the evacuations referred to were a sign, or a consequence, rather than a means of improvement, the latter seems the most probable supposition.

In accounting for the good effect of purgatives we find a probable explanation of the more favorable operation of those of the saline class. The abundant aqueous effusion which they produce, probably taking place chiefly in the upper portion of the alimentary canal, makes them efficient in washing away the contents of the large intestine, with but little drastic effect upon the latter.* It is possible, also, that the effusion may sometimes be useful as a mode of depletion.

Some practitioners have employed large enemas, filling the large intestine with liquid by means of a flexible tube attached to the syringe and carried up to, or above the signoid flexure, in order to effect removal of the contents of the bowel. This must be less effectual than the successful operation of saline purgatives, and the introduction of the tube must be painful, and expose the inflamed surface of the rectum to injury. Theoretically, purgative remedies are to be preferred.

*Mercury.* Calomel is sometimes given as a cathartic in dysentery. It is also frequently prescribed under the supposition that it exerts a remedial influence in this disease, irrespective of its operation as a purgative. With a view thereto it is usually given in combination with opium.

A portion of the cases, on the analysis of which was based the foregoing clinical report, were treated with calomel and opium, and I have endeavored to institute a fair comparison of the cases in which this treatment was pursued with the cases treated without mercury. The results of this comparison, and the conclusions deduced thereon, are given in the report, (see section twelfth.) So far as these cases are concerned, the facts do not afford support to the opinion that mercury exerts any marked remedial influence in dysentery.

Mercury is supposed to possess a power antagonistic to local inflammation

---

* This statement with respect to the operation of saline purgatives is undoubtedly true if this operation depend (as has been maintained by M. Poisseuille, Liebig, Pereira, Golding Bird, and others,) on their causing an *exosmosis* of the watery portion of the blood. This view of the operation of saline purgatives has lately been called in question by Dr. Headland (prize essay on the action of medicines.) Dr. H. thinks that, like other cathartics, (castor-oil, etc.,) they are absorbed into the blood, and afterward eliminated. If so, the aqueous effusion may take place from the large intestine.

wherever situated. Assuming this supposition to be well founded, it is reasonable to presume that the remedy may affect, more or less, the inflammatory element in dysentery. It is possible that the analysis of a larger collection of cases might develop some proof of the correctness of this presumption.

It is, however, believed by some practitioners, that mercury is in a more special manner useful in this disease. This belief is based on pathological notions which would be hardly worthy of notice, were it not that they are recognized in some modern systematic works on practical medicine. The notions referred to are, that dysentery has some dependent connection with suppressed secretion of the liver, and congestion of the portal system of veins; and that mercury, by exciting the secretory action of the liver, relieves the congestion, and, consequently, the dysenteric affection. With respect to this view of the pathology of dysentery, it is only necessary to say that the premises are assumed without proof; and, admitting their correctness, we have no evidence of that connection between them which the reasoning requires. That suppression of bile is one of the primary events in the pathological history of the disease, is not ascertained; that portal congestion is a consequence of such suppression is a mere supposition; and that portal congestion is adequate to the production of dysentery is opposed by the well known consequences of this congestion when it is certain that it does exist.

Fanciful as is this hypothesis, it is the basis of that confidence in the efficacy of mercury in dysentery which has led practitioners, in some parts of this country, to place their chief reliance upon it in the treatment. Fortunately for the success of the treatment, in order better to secure the special operation of the mercury, it is generally given in combination with opium! With preconvictions according to mercury whatever efficacy pertains to the combination of calomel and opium, it is not surprising that experience appears to furnish evidence of the truth of this hypothesis.

*Opium.* From an early period in the history of medicine, opium has entered into the treatment of dysentery. It would be difficult to find any authority in the medical literature of past ages, for the disuse of opium in this disease. The only difference of opinion has related to the degree of reliance to be placed on it, and the liability to injurious effects if given largely. Medical opinions and practice, at the present moment, exhibit the same agreement and differences. Almost every practitioner prescribes opium, to a greater or less extent, in dysentery; some regard it as the chiefly important therapeutical agent in the treatment, and others look upon it as simply adjunctive. It is consequently employed much more freely by some practitioners than by others.

The immediate apparent effect of this remedy is often striking. Sometimes it appears promptly to arrest the disease. Instances of the latter description, however, are not sufficiently numerous to warrant the inference that the remedy has an efficacy which can properly be styled specific. We can readily account for more or less relief of the distressing symptoms of dysentery—the tormina and tenesmus—by the anodyne properties of opium. It is serviceable in another mode, in which, perhaps, consists its chief efficiency, viz., keeping the inflamed part in a state of quietude. It does this by arresting the peristaltic movements, and by relieving the tenesmus so that the patient does not feel impelled to make frequent efforts to evacuate the bowels. The remedy may exert a curative power in other modes which, with our present knowledge, are not understood.

To secure the ends just stated, opium must be given in doses sufficient to produce a decided impression. Experience teaches that the quantity requisite to produce the desired effect differs widely in different persons, and in different diseases, irrespective of pain. This is a very important practical point. A dose of opium which affects one person in a sensible manner, has little or no apparent effect on another person under similar circumstances; and in some diseases in which pain is not a prominent symptom, there is an extraordinary tolerance of this drug. The latter appears to be true of dysentery. Opium may be given, in some cases, at least, of this disease, in large doses without any manifestations of its narcotic properties; and it *must* be given in large doses to secure the desired remedial effects. I have known twenty-four grains of the sulphate of morphia to be taken in twenty-four consecutive hours, by a patient not habituated to the use of opium, with no evidence of narcotism, nor could this quantity be diminished without marked aggravation of the symptoms.

Bearing in mind the differences in susceptibility to the remedy, it is of course highly important to avoid any risk of too great narcotic effect. This is to be done by increasing the doses, by degrees, up to the requisite amount, allowing intervals between them sufficient to estimate the effect of each dose.

Taking into view the *modus operandi* of opium (so far as it is explicable) we can understand why its good effects may be more marked, after free purgation. The intestine is more likely to remain in a quiescent state in proportion as it is free from fecal contents to excite peristaltic movements, and provoke acts of defecation.

The different preparations of opium may be employed according to circumstances. Those which are most prompt in their action are preferable, in order that the effects of each dose may be better estimated. Concentrated preparations are also frequently retained, when those more bulky are rejected by vomiting. The salts of morphia are particularly eligible in cases in which irritability of the stomach exists. Opium in tincture, or aqueous solution, and the salts of morphia, given by enema, often have a very happy effect. Frequently this mode of administration is ineffectual in consequence of the speedy expulsion of the enemas. This is very apt to occur in the course of the disease after their frequent repetition; and sometimes, under these circumstances, to persist in their use is injudicious, the irritability of the rectum being increased by them, and the efforts of defecation rendered more frequent and painful than would otherwise be the case.

Other injections are employed in dysentery. A strong solution of the nitrate of silver sometimes appears to allay the tenesmus, and render the dejections less frequent; in other cases it produces no such effects, and sometimes occasions severe pain. As a local application to the inflamed mucous surface, its action must of course be very limited, especially if the injection be made into the rectum alone. Carried higher up into the intestinal canal by means of a long tube, it may be brought into contact with a larger portion of the affected surface, but even by this mode its action must still be limited. I cannot speak of the latter mode from personal trial of it. Creasote, in mixture, appears, in some instances, to allay the irritability of the rectum. Strong testimony is borne, by numerous practitioners, to the relief afforded by large enemas of simple cold water, repeated more or less frequently.*

*Astringents.* Various astringent medicines are in common use in dysentery, viz., the acetate of lead, the nitrate of silver; and, of vegetable astringents, a great number such as tannic acid, rhatany, kino, catechu, etc. These are very rarely, if ever, relied upon to the exclusion of other remedies, and they are usually given in connection with opium. It is, therefore, difficult to estimate the effect which belongs to them separately. The impression formed from my own experience is, that they are comparatively of small utility. It is desirable that the utility, or non-utility of this class of remedies should be determined, because, in the first place, if useful, it is important not

---

* In a case, coming under observation since this was written, in which hemorrhage from the bowels occurred after the dysenteric symptoms had greatly diminished, enemas of cold water appeared to produce a decided effect in arresting the flow of blood. They were also grateful to the patient.

to deprive ourselves of their aid; and, in the second place, if not useful, although directly they may not be injurious, they are likely to prove so, indirectly, by appropriating a certain portion of that dependence which otherwise we should place on remedies more efficient for good.

A host of remedies might be mentioned which have been recommended in dysentery, and which do not fall within the foregoing classes; such are belladonna, stramonium, aconite, balsam of copaiva, ipecacuanha, nux vomica, etc., etc. I have already disclaimed, for these remarks, a scope and particularity embracing an examination of the evidence in support of the efficacy of these various remedies, or a discussion of their *modus operandi.* I shall therefore pass them over without farther notice.

*Revulsive applications.* These are frequently employed in dysentery. Blisters to the abdomen, and to other parts of the body; sinapisms and stimulating liniments, are thought, by some practitioners, to be highly useful. My experience is limited to sinapisms and liniments. These are evidently subordinate measures, to which not much importance is to be attached in controlling the progress and issue of the disease. Rationally considered, more active counter-irritation is of doubtful expediency.

Local soothing applications, viz., fomentations and cataplasms, belong among the minor adjunctive measures of treatment, having some value.

*Supporting measures.* In cases of dysentery characterized by the extent and intensity of the local affection, the latter tending to disorganization, or ulcerations, and in cases in which, from feebleness of constitution, or other causes, there follow prostration, and a tendency to a fatal issue by asthenia, denoted especially by frequency and feebleness of the pulse, supporting measures become very important. Under such circumstances, after the disease has reached its maximum, these measures, in fact, constitute our main reliance, the indication being to sustain the powers of the system through the processes of restoration.

The supporting measures consist of tonics, diffusible stimulants, and nutriment. Inasmuch as the effects of tonics are too slowly produced to accomplish much within a short period, they are far less important than stimulants and nutriment. I have witnessed very striking apparent effects from the use of spirits in some grave cases of dysentery. In one of the cases in the collection analyzed, in which the pulse remained at 140 for several successive days, it rapidly decreased in frequency, other symptoms at the same time evincing improvement, under the free administration of brandy, the patient being a female wholly unaccustomed to the use of spirituous beverages. In two cases, not embraced in the collection analyzed, in which the disease was graver and more persisting than in any other instances ending in recovery that have ever fallen under my observation, the patients were apparently saved by supporting measures carried out with boldness and perseverance. In one of these cases, the patient, a temperate man, took on the day before there occurred a distinct amelioration of symptoms, twenty-four ounces of brandy, in doses of a half ounce half-hourly. In this case it was observed that if the interval between the doses was prolonged, as it was sometimes in order to observe the result, the pulse became more frequent and feeble. The vital forces were apparently sustained, and the patient carried through the disease by vigorous unremitting stimulant treatment.

The quantity of spirits to be administered in a given time; the doses; the frequency of their repetition, etc., etc., are of course, to be varied in different cases according to circumstances. These are among the details upon which I do not propose now to enter.

The systematic administration of nutriment is an important part of the supporting measures. It is to be given regularly, and by persuasion if the patient be disinclined to it. The kind, and proper preparation of the nutriment are all important. It should be concentrated, *i. e.*, contain as much nutriment in as small bulk as possible, and, considering the seat of the local affection, (the large intestine,) the importance of its being so entirely assimilable as to leave a small quantity of fecal excrement, is obvious. The animal essences, or concentrated broths, with milk, and small proportion of some farinaceous substance, will unite the alimentary principles necessary for combustion and nutrition.

I am convinced that with a due appreciation of the importance of supporting measures, on the part of the practitioner, together with promptness, boldness, and perseverance, in their use, and a careful supervision of all the details which they involve, patients are sometimes carried safely through this disease, when the gravity of the affection is such that a fatal termination would otherwise be inevitable.

To recapitulate, in a few words, some of the more important of the practical points involved in the foregoing considerations: rational principles, and what light is afforded by experimental knowledge, lead us to regard opium as by far the most valuable of the remedies employed in the management of dysentery; but to secure the full efficiency of this remedy, in this disease, it must be given in doses sufficient to fulfill the indications which it is designed to meet, adapting quantity and modes of administration to the peculiarities of individual cases, and, at the same time, observing due precautions in its use. Blood-letting, it is probable, is useful in some instances, but it is to be employed with discrimination, and cautiously graduated by the circumstances which indicate it. Purgatives, especially saline, are frequently important by way of preparation for the good effect to be expected from the use of opium. Astringents, and the numerous remedies supposed to be useful in this disease, may be serviceable to a greater or less extent; but, relatively, they hold the position of auxiliary measures, and their employment should not detract from reliance on those which are more efficient. Finally, after employing means to abate the intensity of the disease, and dispose to its resolution, the chief object of the management in cases which resist these means, is to sustain the vital forces, with the hope of prolonging life, and developing power to carry on and complete the processes of restoration.

